# FOXC2-AS1 regulates phenotypic transition, proliferation and migration of human great saphenous vein smooth muscle cells

**DOI:** 10.1186/s40659-019-0266-z

**Published:** 2019-12-04

**Authors:** Chuang Zhang, Huixiang Li, Xueli Guo

**Affiliations:** 1 Department of Pathology, Basic Medical College of Zhengzhou University, No. 100 Science Avenue, Zhengzhou, 450001 Henan China; 2 Department of Vascular Surgery, The First Affiliated Hospital of Zhengzhou University, Zhengzhou, 450002 Henan China

**Keywords:** Varicose vein, FOXC2-AS1, FOXC2, Notch, Smooth muscle cells

## Abstract

**Objectives:**

In varicose veins, vascular smooth muscle cells (VSMCs) often shows phenotypic transition and abnormal proliferation and migration. Evidence suggests the FOXC2–Notch pathway may be involved in the pathogenesis of varicose veins. Here, this study aimed to explore the role of long non-coding RNA FOXC2-AS1 (FOXC2 antisense RNA 1) in phenotypic transition, proliferation, and migration of varicose vein-derived VSMCs and to explore whether the FOXC2-Notch pathway was involved in this process.

**Methods:**

The effect of FOXC2-AS1 on the proliferation and migration of human great saphenous vein smooth muscle cells (SV-SMCs) was analyzed using MTT assay and Transwell migration assay, respectively. The levels of contractile marker SM22α and synthetic marker osteopontin were measured by immunohistochemistry and Western blot to assess the phenotypic transition.

**Results:**

The human varicose veins showed thickened intima, media and adventitia layers, increased synthetic VSMCs, as well as upregulated FOXC2-AS1 and FOXC2 expression. In vitro assays showed that FOXC2-AS1 overexpression promoted phenotypic transition, proliferation, and migration of SV-SMCs. However, the effect of FOXC2-AS1 overexpression could be abrogated by both FOXC2 silencing and the Notch signaling inhibitor FLI-06. Furthermore, FOXC2-AS1 overexpression activated the Notch pathway by upregulating FOXC2.

**Conclusion:**

FOXC2-AS1 overexpression promotes phenotypic transition, proliferation, and migration of SV-SMCs, at least partially, by activating the FOXC2-Notch pathway.

## Background

Lower extremity varicose veins are a common disorder of venous dilation and tortuosity, and most varicose veins occur in the great saphenous vein [1, 2]. The phenotypic transition of vascular smooth muscle cells (VSMCs) and the consequently increased proliferation and migration are common pathophysiological processes of vascular remodeling-related diseases including varicose veins [3]. Under normal circumstances, VSMCs mainly express contractile phenotype and maintain the elasticity of the blood vessel walls and regulate blood flow; while in response to vascular injury or pathological conditions, VSMCs can experience transition from a contractile to synthetic phenotype (i.e. “phenotypic transition”) and subsequently maintain increased proliferation and migration, thereby leading to vascular remodeling [2].

The forkhead box C2 (FOXC2) is a transcription factor of the human forkhead family involved in the metabolism of adipose cells, and is closely related to the occurrence and development of blood vessels and lymphatic vessels [4, 5]. Notably, FOXC2 is one of the pathogenic genes most closely associated with the developmental defects and dysfunction of venous valves of the lower extremity [6–8]. Evidence indicates that FOXC2 overexpression in venous endothelial cells upregulates the expression of Notch pathway-related proteins (Dll4 and Hey2) [9]. The Notch pathway plays a key role in the development of vascular networks [10]. These findings collectively suggest the involvement of FOXC2-Notch pathway in the pathogenesis of varicose veins.

Long non-coding RNAs (lncRNAs) have been shown aberrantly expressed in the primary great saphenous varicose veins, suggesting the potential involvement of lncRNAs in the pathogenesis of varicose veins [11]. FOXC2 antisense RNA 1 (FOXC2-AS1) is a recently discovered lncRNA. Several studies have uncovered the pro-tumorigenic role of FOXC2-AS1 in cancers [12, 13]. However, the exact role of FOXC2-AS1 in varicose veins remains unclear. Evidence has indicated that the expression of FOXC2 can be regulated by its antisense lncRNA FOXC2-AS1 that can form a double-stranded structure with FOXC2 mRNA and promotes the stability of FOXC2 mRNA [14]. These findings indicate that FOXC2-AS1 might be involved in the pathogenesis of varicose veins.

In this study, we first investigated the differences in morphology, VSMCs phenotypic transition, and FOXC2-AS1 expression between the normal veins and varicose veins. Subsequently, we investigated the role of FOXC2-AS1 in regulating phenotypic transition, proliferation, and migration of human great saphenous vein smooth muscle cells (SV-SMCs). Finally, we elucidated whether the mechanisms underlying the FOXC2-AS1-mediated effect was related to the regulation of FOXC2 and Notch signaling pathway.

## Results

### Varicose veins show thickened intima, media, and adventitia, as well as increased synthetic smooth muscle cells

**Fig. 1:**
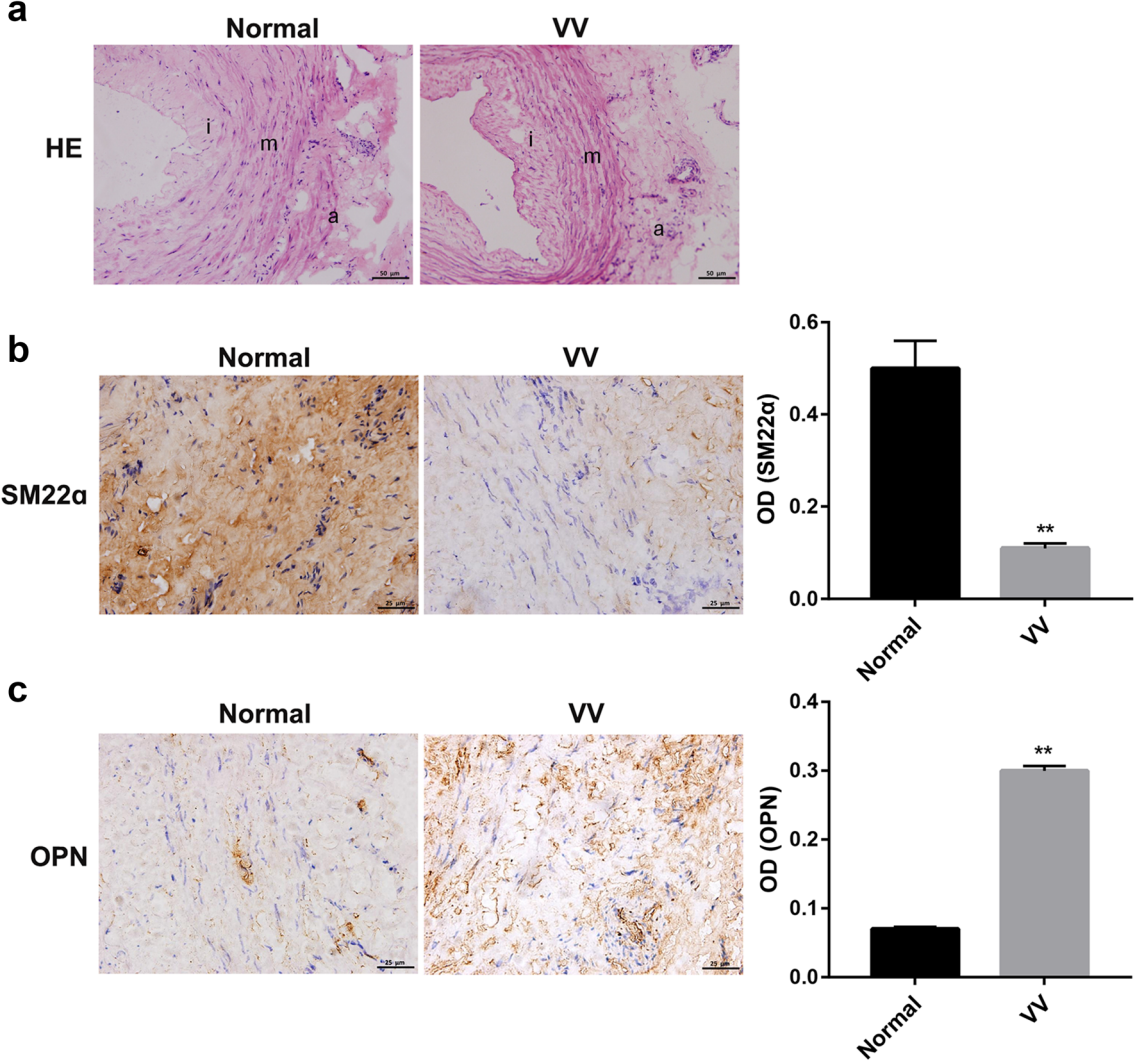
Varicose veins show thickened intima, media and adventitia, as well as increased synthetic smooth muscle cells. **a** HE staining was performed to observe the morphological differences between human varicose veins and normal veins. Scale bar: 50 μm. *i* intima, *m* media, *a* adventitia. **b**–**c** Immunohistochemistry was used to observe the localization and expression of the contractile marker SM22α (**b**) and the synthetic marker OPN (**c**) in human varicose veins and normal veins. The mean optical density (OD) was measured using Image-Pro Plus 6.0 software. Scale bar: 25 μm. N = 10/group. *Normal* normal veins, *VV* varicose veins

HE staining showed that the thickness of the intima, media, and adventitia of normal veins was normal. However, in the varicose veins, abnormal thickening of the intima was observed, and VSMCs proliferated in the media and adventitia of veins (Fig. 1a). Immunohistochemistry analysis revealed SM22α-positive signals and almost no osteopontin (OPN)-positive cells in the normal veins. However, in the varicose veins, SM22α protein staining showed a weak positive signal, whereas OPN was widely distributed in the smooth muscle cells of the neointima of the vessel walls (Fig. 1b, c).

### Varicose veins show upregulated FOXC2-AS1 and FOXC2 expression

**Fig. 2:**
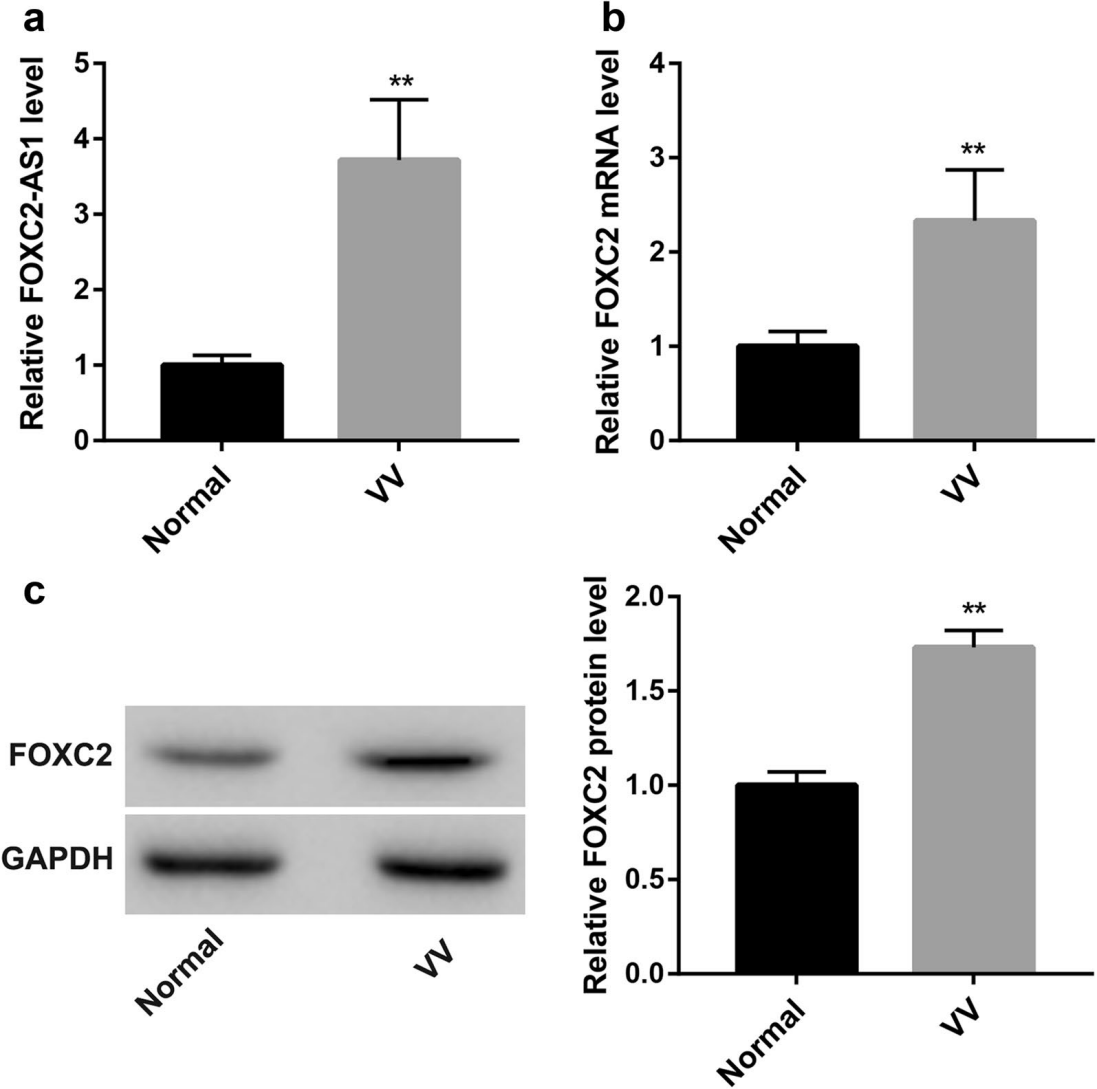
Varicose vein tissues show upregulated FOXC2-AS1 and FOXC2. **a** qRT-PCR was performed to examine the expression of FOXC2-AS1 in human varicose veins and normal veins. The mRNA (**b**) and protein expression (**c**) of FOXC2 in human varicose veins and normal veins were detected by qRT-PCR and Western blot, respectively. GAPDH was used as the loading control. N = 10/group. *Normal* normal veins, *VV* varicose veins. ^**^p < 0.01 vs. Normal group

The qRT-PCR results showed that FOXC2-AS1 expression in the varicose veins was significantly higher than that in the normal veins (Fig. 2a). Furthermore, the mRNA (Fig. 2b) and protein levels (Fig. 2c) of FOXC2 in the varicose veins were also significantly higher compared with the normal veins.

### FOXC2-AS1 overexpression promotes phenotypic transition, proliferation, and migration of SV-SMCs

**Fig. 3:**
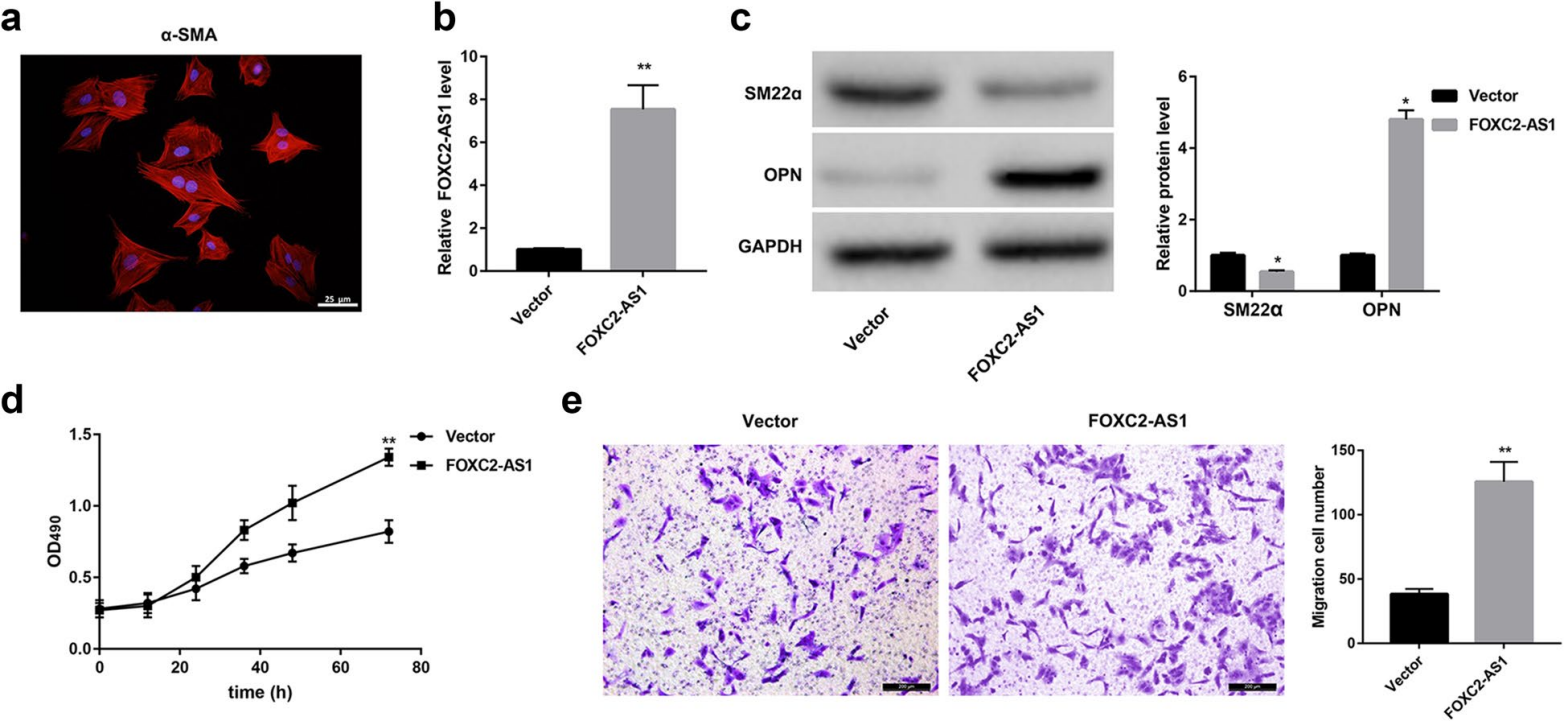
FOXC2-AS1 overexpression promotes phenotypic transition, proliferation, and migration of SV-SMCs. **a** The human SV-SMCs were isolated from normal human great saphenous vein, and then identified by α-SMA immunofluorescence. Scale bar: 25 μm. Red signals indicate α-SMA; blue signals indicate Hoechst 33,342-stained nuclei. **b** The FOXC2-AS1 overexpression vector and empty control were constructed and transfected into SV-SMCs. The overexpression efficiency was detected by qRT-PCR. **c** Western blot was performed to detect the levels of SM22α and OPN. **d** MTT was performed to assess cell proliferation. **e** Transwell migration assays were performed to assess cell migration. Scale bar: 200 μm. ^*^p < 0.05, ^**^p < 0.01 vs. Vector group

We next explored the effect of FOXC2-AS1 overexpression on phenotypic transition, proliferation, and migration of SV-SMCs. The SV-SMCs were confirmed by α-SMA immunofluorescence (Fig. 3a). The overexpression efficiency was confirmed by qRT-PCR (Fig. 3b). Western blot analysis showed that FOXC2-AS1 overexpression significantly downregulated protein levels of the contractile marker SM22α, whereas upregulated levels of the synthetic marker OPN in SV-SMCs. This suggests that FOXC2-AS1 overexpression promotes the transition of SV-SMCs from contractile to synthetic phenotype (Fig. 3c). Furthermore, MTT assay revealed that FOXC2-AS1 overexpression significantly promoted the proliferation of SV-SMCs (Fig. 3d). Moreover, Transwell migration assays showed that FOXC2-AS1 overexpression significantly promoted the migration ability of SV-SMCs (Fig. 3e).

### FOXC2-AS1 overexpression promotes phenotypic transition, proliferation, and migration of SV-SMCs through upregulating FOXC2

**Fig. 4:**
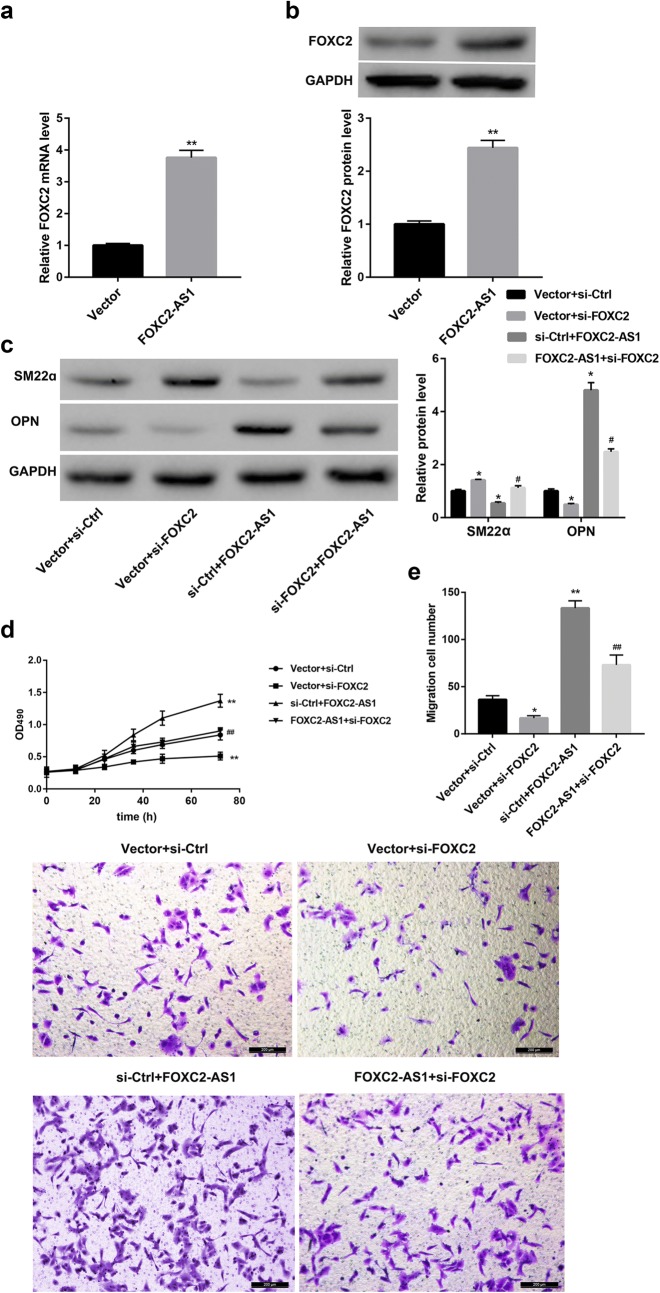
FOXC2-AS1 overexpression promotes phenotypic transition, proliferation and migration of SV-SMCs through upregulating FOXC2. The SV-SMCs were transfected with FOXC2-AS1 overexpression vector and empty vector control, and the mRNA (**a**) and protein expression (**b**) of FOXC2 was detected by qRT-PCR and Western blot, respectively. ^**^p < 0.01 vs. Vector group. SV-SMCs were co-transfected with FOXC2-AS1 overexpression vector and FOXC2 siRNA (si-FOXC2). **c** Western blot was performed to detect the protein levels of SM22α and OPN. **d** MTT assay was performed to detect cell proliferation, and **e** Transwell migration assay was performed to detect cell migration. Scale bar: 200 μm. ^*^p < 0.05, ^**^p < 0.01 vs. Vector + si-Ctrl group. ^#^p < 0.05, ^##^p < 0.01 vs. si-Ctrl + FOXC2-AS1 group

We next elucidated whether FOXC2 involved in the FOXC2-AS1-mediated effect in SV-SMCs. FOXC2-AS1 overexpression upregulated the mRNA (Fig. 4a) and protein levels (Fig. 4b) of FOXC2 in SV-SMCs. Furthermore, FOXC2-AS1 overexpression significantly promoted the transition from contractile to synthetic phenotype (Fig. 4c), proliferation (Fig. 4d) and migration (Fig. 4e) of the SV-SMCs, and this effect was effectively reversed by FOXC2 silencing (Fig. 4c–e). These results suggest that FOXC2-AS1 overexpression promotes phenotypic transition, proliferation, and migration of the SV-SMCs, at least partially, by upregulating FOXC2 expression.

### FOXC2-AS1 overexpression activates the Notch pathway by upregulating FOXC2

**Fig. 5:**
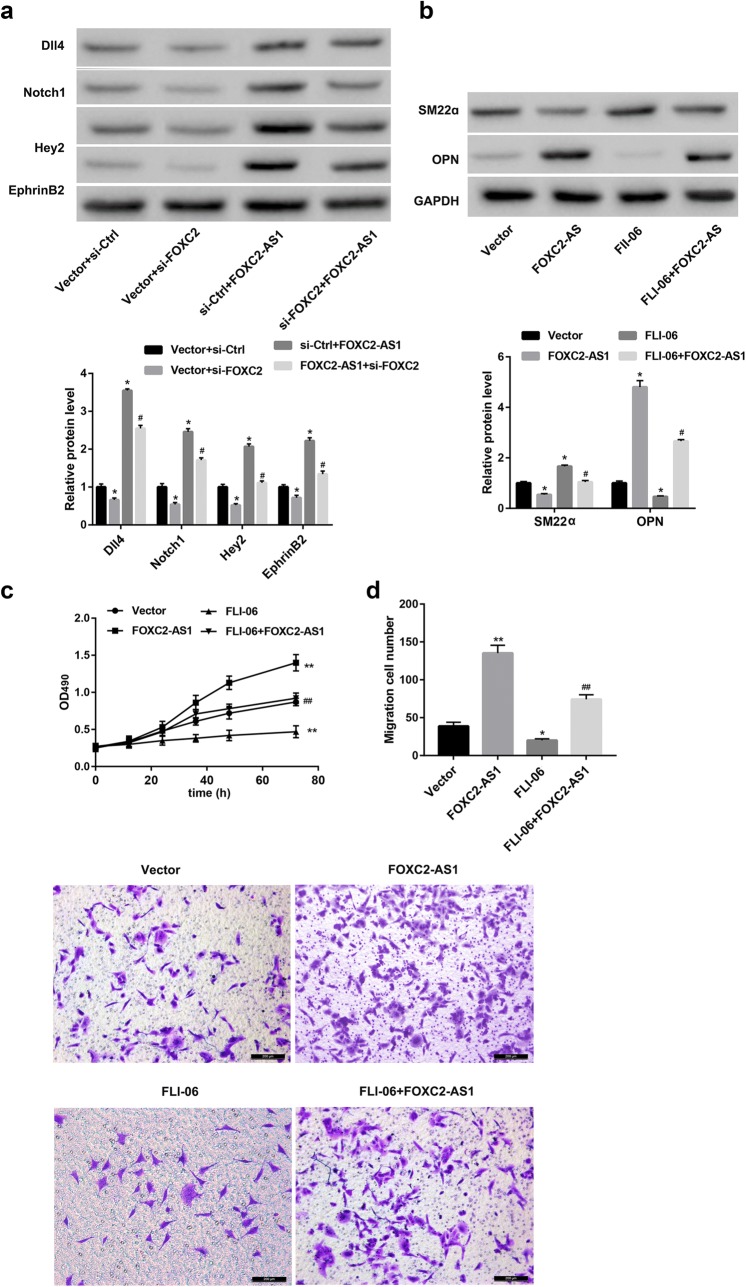
FOXC2-AS1 overexpression promotes phenotypic transition, proliferation, and migration of SV-SMCs through activating Notch pathway. **a** SV-SMCs were co-transfected with FOXC2-AS1 overexpression vector and FOXC2 siRNA (si-FOXC2). Western blot was performed to detect the protein levels of the Notch pathway-related proteins including Dll4, Notch1, Hey2 and EphrinB2. ^*^p < 0.05 vs. Vector + si-Ctrl group. ^#^p < 0.05 vs. si-Ctrl + FOXC2-AS1 group. SV-SMCs were transfected with FOXC2-AS1 overexpression vector and empty vector control, followed by treatment with Notch signaling pathway inhibitor FLI-06. **b** Western blot was performed to detect the protein levels of SM22α and OPN. **c** MTT assay was performed to detect cell proliferation, and **d** Transwell migration assay was performed to detect cell migration. Scale bar: 200 μm. ^*^p < 0.05, ^**^p < 0.01 vs. Vector group. ^#^p < 0.05, ^##^p < 0.01 vs. FOXC2-AS1 group

As shown in Fig. 5a, FOXC2-AS1 overexpression increased, whereas FOXC2 silencing decreased protein levels of the Notch pathway-related proteins including Dll4, Notch1, Hey2 and EphrinB2. Of note, FOXC2 silencing effectively attenuated the FOXC2-AS1 overexpression-mediated increase in protein levels of Dll4, Notch1, Hey2 and EphrinB2. These results suggest that FOXC2-AS1 overexpression activates the Notch pathway by upregulating FOXC2.

### FOXC2-AS1 overexpression promotes phenotypic transition, proliferation and migration of SV-SMCs through activating Notch pathway

Finally, we determined whether the Notch pathway involved in the FOXC2-AS1-mediated effect in SV-SMCs. FOXC2-AS1 overexpression promoted the phenotypic transition (Fig. 5b), proliferation (Fig. 5c) and migration (Fig. 5d) of the SV-SMCs, and this effect can be impaired by Notch pathway inhibitor FLI-06 (Fig. 5b–d). These results suggest that FOXC2-AS1 overexpression promotes phenotypic transition, proliferation, and migration of the SV-SMCs, at least partially, by activating the Notch pathway.

## Discussion

The results in this study showed thickened intima, media and adventitia, and phenotypic transition from contractile to synthetic phenotype of smooth muscle cells in the varicose veins. Importantly, this study provided the first evidence that FOXC2-AS1 expression in the varicose veins was significantly higher than that in the normal veins. Furthermore, in vitro assays revealed that FOXC2-AS1 overexpression promoted the transition from contractile to a synthetic phenotype, proliferation, and migration of the SV-SMCs, at least in part, by upregulating FOXC2 expression and subsequently activating the Notch pathway.

Accumulating evidence has already shown that vascular remodeling plays a crucial role in the pathogenesis of varicose veins. The phenotypic transition of VSMCs from the contractile to synthetic phenotype increases proliferation and migration of VSMCs, thereby leading to vascular remodeling [2]. Hyperplasia of VSMCs in the vein wall and disorders in the arrangement of VSMCs are involved in the development of varicose veins [15, 16]. As expected, our results in this study showed thickened intima, media and adventitia in the varicose veins. Furthermore, a phenotypic transition from contractile to synthetic phenotype of smooth muscle cells was observed in the varicose veins, indicating the involvement of VSMCs dedifferentiation in the pathogenesis of varicose veins.

Evidence has shown that lncRNAs are differentially expressed in the primary great saphenous varicose veins, suggesting that lncRNAs might be involved in the pathogenesis of varicose veins [11]. Several studies have uncovered the pro-tumorigenic role of FOXC2-AS1 in cancers [12, 13]. For example, FOXC2-AS1 has been shown to promote doxorubicin resistance in osteosarcoma [14]. Another study has suggested that FOXC2-AS1 predicts poor survival in breast cancer patients and promotes cell proliferation [12]. Additionally, FOXC2-AS1 facilitates the proliferation and progression of prostate cancer via targeting miR-1253/EZH2 [13]. Recent studies also showed that FOXC2-AS1 protects cardiomyocytes from doxorubicin-induced cardiotoxicity through activation of WNT1-inducible signaling pathway protein-1 [17]. The results in this study showed that FOXC2-AS1 expression was significantly higher in the varicose veins than that in the normal veins, indicating that FOXC2-AS1 may be involved in the pathogenesis of varicose veins.

Studies have shown that the factors regulating the phenotypic transition, proliferation, and migration of VSMCs may be involved in the pathogenesis of varicose veins. For example, the low expression of lncRNA GAS5 facilitates proliferation and migration of SV-SMCs and thereby the pathogenesis of great saphenous veins varicosities [18]. miR-202 was upregulated in varicose veins and proliferative VSMCs. Furthermore, miR-202 induced VSMCs proliferation and migration as well as phenotypic transition, and thereby may act as a novel target for varicose vein therapy [19]. The results in this study indicated that FOXC2-AS1 overexpression significantly downregulated the contractile marker SM22α and upregulated the synthetic marker OPN in SV-VSMCs. Downregulation of VSMCs contractile markers and upregulation of synthetic markers mark the transition of VSMCs from contractile to a synthetic phenotype. Thus, our results suggest that FOXC2-AS1 overexpression promotes the phenotypic transition of SV-SMCs. In addition, FOXC2-AS1 overexpression significantly promoted the proliferation and migration of SV-VSMCs. Accordingly, these results collectively suggest that FOXC2-AS1 may serve as a novel target for varicose vein therapy.

FOXC2 is one of the first pathogenic genes most closely associated with the developmental defects and dysfunction of the primary superficial venous valves of the lower extremity [6–8]. Studies have shown that the expression of FOXC2 can be regulated by its antisense lncRNA FOXC2-AS1 that FOXC2-AS1 can form a double-stranded structure with FOXC2 mRNA and promotes the stability of FOXC2 mRNA [14]. The results here showed that the mRNA and protein levels of FOXC2 in the varicose veins were significantly higher compared with the normal veins. This is consistent with the high expression of FOXC2-AS1 in varicose veins. In addition, FOXC2-AS1 overexpression promotes the phenotypic transition, proliferation, and migration of SV-SMCs by upregulating FOXC2 expression.

The Notch signaling pathway plays a key role in the development and stabilization of blood vessels and is closely associated with the development of vascular networks [10]. Many studies have also shown that the Notch pathway is involved in the regulation of proliferation and migration of VSMCs [20–23]. Importantly, evidence has indicated that FOXC2 overexpression in venous endothelial cells upregulates the expression of Notch signaling pathway-related proteins including Dll4 and Hey2, suggesting that the FOXC2-Notch pathway is associated with varicose veins [9]. Our results here showed that FOXC2-AS1 promotes the phenotypic transition, proliferation, and migration of SV-SMCs by activating the Notch pathway. Furthermore, FOXC2-AS1 overexpression activates the Notch pathway through upregulating FOXC2.

## Conclusion

In conclusion, FOXC2-AS1 overexpression promoted the transition from contractile to synthetic phenotype, proliferation and migration of the SV-SMCs, at least in part, by upregulating FOXC2 expression and subsequently activating the Notch pathway. Our findings provide new insight into the molecular mechanism underlying the pathogenesis of varicose veins and support the possibility of FOXC2-AS1 as a novel target for further treatment for varicose veins.

## Materials and methods

### Sample collection

The varicose veins specimens were collected from 10 patients undergoing lower-extremity varicose vein excision in the First Affiliated Hospital of Zhengzhou University. All tissues were taken from the most obvious portions. Healthy great saphenous vein segments were obtained from 10 patients undergoing cardiac coronary artery bypass grafting surgery. The human experiment was approved by the Ethics Committee of the First Affiliated Hospital of Zhengzhou University.

### Hematoxylin and eosin (HE) staining

HE staining was performed to observe the morphological differences between varicose veins and normal veins. Briefly, the veins specimens were fixed in 10% formalin solution, dehydrated with a graded series of ethanol, infiltrated with xylene, and then embedded in paraffin before being cut into 4-μm thick sections. The sections were stained with HE following the routine staining procedure and analyzed with an Olympus BH-2 light microscope (Olympus, Tokyo, Japan).

### Immunohistochemistry

Immunohistochemistry was used to observe the localization and expression of SM22α and OPN in varicose veins and normal veins. Briefly, the veins sections were dewaxed, hydrated, and then incubated in 3% H_2_O_2_ to quench endogenous peroxidase activity. Subsequently, the sections were rinsed with distilled water three times and then completely immersed in 0.01 mol/L citrate buffer, heated in a microwave oven, and washed twice with PBS. Following incubation with 5% BSA blocking solution, the sections were incubated with a primary rabbit anti-human SM22α (1:200; Abcam) or rabbit anti-human OPN (1:100; Abcam) at 37 °C for 90 min, followed by the biotin-labeled secondary antibodies at 37 °C for 20 min. The sections were incubated with the streptavidin–biotin–peroxidase complex (SABC) working solution at 37 °C for 20 min and then washed with PBS containing 0.1% TWEEN20. Then samples were stained with diaminobenzidine (DAB), counterstained with hematoxylin, dehydrated, and then embedded in paraffin. The yellowish-brown staining indicates a positive signal. The sections were observed under an Olympus BH-2 light microscope (Olympus, Tokyo, Japan).

### Quantitative real-time PCR (qRT-PCR)

qRT-PCR was performed to examine the expression of FOXC2-AS1 and FOXC2. Total RNA was extracted from tissues or cells using TRIzol reagent (Invitrogen) and was reverse transcribed into cDNAs using the Reverse Transcription Kit (Takara). The cDNA template was synthesized through qRT-PCR using SYBR Green PCR Kit (Thermo Fisher Scientific, Waltham, MA, USA) by the ABI7900 system (Applied Biosystem). The relative mRNA expression levels were calculated by the 2^−ΔΔCt^ method and normalized to GAPDH. The primers were as follows: FOXC2-AS1 (forward) 5′-TTCATCGGCTGCGTATTCG-3′, FOXC2-AS1 (reward) 5′-TTGCCTTCTAGTCGCCTCC-3′; FOXC2 (forward) 5′-CGGCCCAGCAGCAAACTTTCC-3′, FOXC2 (reward) 5′-AGAGGCGGCGTGGATCTGTAG-3′; GAPDH (forward) 5′-CGCTGAGTACGTCGTGGAGT-3′, GAPDH (reward) 5′-CGTCAAAGGTGGAGGAGTGG-3′.

### Isolation and culture of human SV-SMCs

The human SV-SMCs were isolated from healthy human great saphenous vein. Briefly, the saphenous veins from the patients undergoing cardiac coronary artery bypass grafting surgery were collected and washed with PBS to remove blood. Then the extravascular connective tissues were carefully removed and the adventitia was exfoliated. The blood vessels were cut longitudinally and the inner membranes were bluntly scraped with the scalpel’s knife back. After being digested in 0.25% trypsin–EDTA solution for 5 min, the middle layer of the blood vessel was cut into a tissue block of about 1 mm × l mm × 1 mm in DMEM medium. Subsequently, the tissue block was evenly spread using an elbow pipette at a density of 3–5 pieces/cm^2^ in a 25 mL glass culture flask, after which the flask was then inverted to stand upright and cultured at 37 °C. When the tissue block and the bottom of the bottle were observed to be firmly attached, the flask was placed flat, and then the complete medium (DMEM medium containing 10% fetal bovine serum, 100 mg/L streptomycin, and 100 U/mL penicillin) was added. After incubation for 12–14 days, the cells are crawled out from the edge of the tissue block, fused, and were routinely digested and passaged. The medium was changed once every 2 days.

### Identification of human SV-SMCs

The SV-SMCs of the 3–5th generation were harvested and identified by α-SMA immunofluorescence. Briefly, when the cell confluence reached 75–85%, cells were fixed with 4% paraformaldehyde, permeabilized in 0.3% Triton X-100, and blocked with normal goat serum. Cells were then incubated with primary anti-α-SMA (1:110) overnight at 4 °C, followed by Alexa Fluor 568-labeled secondary antibody (1:60) at 37 °C for 1 h. Then cells were stained with Hoechst 33,342 and observed under a fluorescence microscope.

### Cell transfection

To overexpress FOXC2-AS1, the full-length FOXC2-AS1 cDNA fragments were cloned into the pcDNA3.1 plasmid (Invitrogen), generating pcDNA3.1-FOXC2-AS1. An empty pcDNA3.1 vector was used as the control. To knock down FOXC2, FOXC2 siRNA (si-FOXC2) was designed and synthesized by GenePharma (Shanghai, China). A scramble siRNA was used as negative control (si-Ctrl). The SV-SMCs were transfected with these constructs using Lipofectamine^®^ 2000 (Invitrogen) following the manufacturer’s protocol.

### Western blot

Total protein from veins tissues and SV-SMCs was extracted using RIPA buffer (Beyotime, China). The protein concentrations were determined by BCA assay. Subsequently, equal protein from cell lysates was separated by 10% SDS-PAGE gels and transferred to PVDF membranes (Millipore Corp., Billerica, MA, USA). After blocked with 5% skim milk, the membranes were incubated with the following primary antibodies against SM22α, OPN, FOXC2, Dll4, Notch1, Hey2, and EphrinB2 (all from Abcam) overnight at 4 °C, followed by the horseradish peroxidase (HRP)-conjugated secondary antibodies (1:1000; Abcam) at room temperature for 1 h. The protein was detected with an enhanced chemiluminescence kit (YEASEN, Shanghai, China) and the band intensity was quantified with Image J 14.0 software. GAPDH served as the loading control.

### Cell proliferation assay

Cell proliferation was analyzed by the MTT assay. Briefly, the SV-SMCs were harvested for 48 h after transfection and then seeded into 96-well plates at a density of 2 × 10^3^–5 × 10^3^ cells/well. After 48 h of incubation, 20 μL MTT (Sigma, 5 mg/mL in PBS) was added into each well for 4 h of incubation at 37 °C. Then the medium was replaced with 150 μL DMSO (Sigma) for 10 min. Cellular viability was determined by measuring the optical density (OD) at 490 nm with averages from triplicate wells by an enzyme-labeled analyzer. Cellular viability was normalized to control well.

### Transwell migration assay

Transwell assays were performed to assess cell migration. Briefly, the transfected cells were resuspended in serum-free medium, and 100 μL of the cell suspension was seeded in the upper chamber of Transwell inserts, while 600 μL full-serum medium containing DMEM with 10% FBS was added to lower chambers. After 24 h of incubation, non-migratory cells on the upper chamber of the inserts were scraped off with a cotton swab. Cells cling to the bottom side were fixed with methanol and stained with 0.1% crystal violet. The number of migratory cells was counted in six random fields under a microscope.

### Statistical analysis

All statistical analyses were performed using SPSS 19.0. The data are presented as the mean ± standard deviation. The unpaired Student’s *t*-test was used to analyze differences between two groups. One-way ANOVA was used to analyze differences among multiple groups. p < 0.05 was considered to indicate statistically significant.

## Data Availability

The datasets used and/or analysed during the current study are available from the corresponding author on reasonable request.

## Abbreviations

DAB: diaminobenzidine
FOXC2: forkhead box C2
FOXC2-AS1: FOXC2 antisense RNA 1
HE: hematoxylin and eosin
HRP: horseradish peroxidase
lncRNAs: long non-coding RNAs
OD: optical density
qRT-PCR: quantitative real-time PCR
SABC: streptavidin–biotin–peroxidase complex
si-FOXC2: FOXC2 siRNA
SV-SMCs: saphenous vein smooth muscle cells
VSMCs: vascular smooth muscle cells
